# Research Progress on Icariin Promoting Bone Injury Repair and Regeneration

**DOI:** 10.3390/ph18081174

**Published:** 2025-08-08

**Authors:** Weijian Hu, Yameng Si, Xin Xie, Jiabin Xu

**Affiliations:** 1 Medical College, Northwest University, Xi’an 710127, China; 202324229@stumail.nwu.edu.cn; 2 School of Stomatology, Xuzhou Medical University, Xuzhou 221004, China; 18905200972@189.cn; 3 College of Life Sciences, Northwest University, Xi’an 710127, China

**Keywords:** icariin, bone regeneration, signaling pathways, anti-inflammatory, angiogenesis, multi-organ protection, pharmacokinetics, multi-target effects

## Abstract

Icariin (ICA) is a bioactive flavonoid compound extracted from *Epimedium* plants. In recent years, it has attracted significant research interest in the field of bone tissue repair due to its pharmacological effects via multiple targets and pathways. Studies have shown that ICA promotes the osteogenic differentiation of mesenchymal stem cells (MSCs) and enhances bone matrix formation by regulating signaling pathways such as Akt and Wnt/β-catenin. It concurrently inhibits osteoclast activity to maintain the balance of bone remodeling, thereby simultaneously stimulating new bone regeneration and suppressing bone resorption. At the same time, ICA exerts potent anti-inflammatory and antioxidant effects and promotes angiogenesis, improving the local microenvironment of bone injury and significantly facilitating the regeneration of bone and cartilage tissues. Additionally, ICA exhibits notable protective effects in multiple organ systems including the cardiovascular, hepatic, renal, and nervous systems. Specifically, ICA reduces cardiomyocyte apoptosis and fibrosis to preserve cardiac function, improves hepatic metabolic function and alleviates oxidative stress, attenuates renal inflammation and fibrosis, and—through neuroprotective actions—reduces neuroinflammation and promotes neuronal survival. These multi-organ effects help optimize the systemic environment for bone healing. However, ICA faces significant pharmacokinetic challenges. It has low oral bioavailability (due to poor absorption and extensive first-pass metabolism) as well as a short half-life. Consequently, maintaining effective drug concentrations in vivo is difficult, which limits its therapeutic efficacy and impedes clinical translation. To fully realize its regenerative potential, advanced drug delivery strategies (e.g., nanocarrier-based delivery systems) are being explored to enhance ICA’s bioavailability and prolong its duration of action. Overall, ICA’s multi-modal actions on bone cells, the immune microenvironment, and systemic factors make it a promising multi-target agent for bone regeneration. Addressing its pharmacokinetic limitations through optimized delivery and conducting further clinical studies will be crucial to realize its full therapeutic potential. This review provides a comprehensive overview of recent advances and challenges in translating ICA’s benefits into orthopedic therapy.

## 1. Introduction

### 1.1. Clinical Challenges of Osteochondral Injuries

Bone and cartilage tissues are core components of the musculoskeletal system, and injuries to these tissues have a high incidence in clinical practice. Such injuries are commonly seen in trauma, age-related degeneration, or metabolic disorders, and in severe cases, they can lead to joint dysfunction and decreased quality of life. Although regenerative medicine and tissue engineering have made significant advances in recent years in repair materials, bioactive factors, and stem cell applications, the repair of complex structural regions like the osteochondral interface and combined cartilage–bone matrix defects remains a technical bottleneck in clinical practice [1].

Osteochondral defects (OCDs) can result from chronic degenerative conditions such as osteoarthritis or osteochondritis or from acute traumatic injury. Their main pathological features include degeneration of hyaline cartilage, destruction of subchondral bone, activation of inflammatory responses, and diminished joint load-bearing capacity, with particularly high incidence in middle-aged and elderly populations [2]. Current mainstream surgical treatments, such as microfracture, osteochondral autograft transplantation, or mosaicplasty, can alleviate pain and improve function in the short term. However, the repair tissue is mostly fibrocartilage dominated by type I collagen, which cannot fully replace the native type II collagen structure of hyaline cartilage; the repaired tissue lacks sufficient mechanical strength and wear resistance, leading to suboptimal long-term outcomes. Interventions for bone defects, such as autologous or allogeneic bone grafts, can provide bone substitute material, but their limitations include donor site morbidity, immune rejection, and infection risks. Total joint replacement (TJR) can address end-stage joint disease, but its use is mainly limited to severe cases or older patients, and complications such as prosthesis loosening, foreign-body reactions, and the need for revision surgery remain unsolved clinical challenges. Therefore, developing a safe, controllable, and regenerative strategy for osteochondral repair remains one of the major challenges in orthopedics [3].

### 1.2. Osteoporosis and Its Relationship with Metabolic Disorders and Secondary Diseases

Osteoporosis is a common systemic skeletal disease characterized by decreased bone mineral density (BMD) and compromised bone microarchitecture, which culminates in weakened mechanical integrity and a markedly increased fracture risk. Given its asymptomatic onset, the condition often goes undiagnosed until a low-energy fracture manifests, earning it the designation of a “silent disease.” According to WHO guidelines, diagnostic criteria are based on DXA-derived T-scores, with values below −2.5 indicating osteoporosis and −1.0 to −2.5 defined as osteopenia [4]. Fragility fractures represent the most serious clinical outcome, significantly impairing quality of life and burdening healthcare systems [5]. Among postmenopausal women, estrogen deficiency—a hallmark of menopause around age 51—is a primary contributor to rapid bone loss through enhanced osteoclast activity, impaired calcium regulation, and compromised bone mechanosensitivity. This hormonal shift may lead to a 12% decrease in bone mass within 5–7 years, along with trabecular thinning and cortical porosity [6]. In secondary osteoporosis, such as glucocorticoid-induced osteoporosis (GIOP), bone quality deterioration rather than BMD alone predicts fracture risk. Glucocorticoids inhibit osteoblastogenesis and alter bone remodeling, complicating risk assessment especially in younger patients on long-term steroid therapy. These limitations necessitate proactive intervention strategies in at-risk populations [7]. Diabetes mellitus, both type 1 and type 2, further exemplifies how chronic metabolic disease can undermine skeletal integrity through mechanisms including impaired osteoblast function, accumulation of advanced glycation end-products (AGEs), altered remodeling, vascular complications, and muscle weakness. While type 1 diabetes is associated with reduced BMD, type 2 diabetes paradoxically presents with normal or elevated BMD but greater fracture risk, implicating compromised bone quality as the key determinant [8]. The gut microbiota has recently been recognized as a critical regulator of bone health via the gut–bone axis, wherein microbial metabolites modulate IGF-1 expression and the release of gut hormones like GLP-1, GLP-2, and peptide YY. Dysbiosis and intestinal barrier dysfunction promote chronic inflammation, disrupting osteocyte viability and skeletal homeostasis [9]. Experimental evidence links high-fat diets and metabolic syndrome to altered gut microbiota and bone loss, while probiotic interventions in estrogen-deficient models have demonstrated protective effects on bone density, underscoring the microbiota’s role in postmenopausal osteoporosis [10]. Furthermore, several endocrine and metabolic disorders—including CKD-MBD, Cushing’s syndrome, hyperthyroidism, and hypogonadism—exert deleterious effects on bone through distinct pathophysiological pathways such as mineral imbalance, hormonal dysregulation, Wnt signaling inhibition, and apoptosis of osteoblasts. Comprehensive management of these conditions is fundamental to the prevention of secondary osteoporosis [11]. In the elderly, osteoporosis often coexists with metabolic comorbidities such as obesity, type 2 diabetes, NAFLD, dyslipidemia, and cardiovascular disease. Although partly attributable to aging, shared molecular pathways may underlie this intersection. Some anti-osteoporotic agents exhibit metabolic benefits, while medications for metabolic syndromes may adversely affect skeletal health. Therefore, individualized therapeutic planning that accounts for a patient’s metabolic profile is essential to optimize outcomes and minimize iatrogenic harm, particularly in postmenopausal women [12].

### 1.3. Biological Potential of Icariin and Research Background

In the context of complementary and alternative medicine (CAM) becoming integrated into mainstream healthcare, natural products with low toxicity, multi-target actions, and good biocompatibility have gained increasing attention for their potential in degenerative bone and joint diseases [13]. As a classic traditional Chinese medicinal herb, *Epimedium* has long been used under the theory that “the kidney governs bones to produce marrow” to treat conditions like osteoporosis, weakness of the limbs, and rheumatism. Its main active constituent—icariin (ICA)—with a well-defined chemical structure, known bioactivity, and clinical safety, has become a focus of research in recent years [14]. ICA is a natural flavonoid compound with a stable structure, primarily found in *Epimedium* species. It has demonstrated favorable bio-pharmacological activities in numerous in vitro and in vivo experiments, including inflammation modulation, alleviation of oxidative stress, and induction of stem cell osteogenesis, which provide an experimental foundation for its potential application in bone tissue repair [15]. Early studies centered on its anti-aging and aphrodisiac effects, whereas in recent years research has shifted toward bone tissue regeneration, osteoporosis prevention, and cartilage repair [16].

In terms of bone metabolism, ICA can promote the differentiation of mesenchymal stem cells (MSCs) into osteoblasts, enhance their mineralization activity and bone matrix deposition rate, thereby significantly increasing bone formation in various bone defect models [17]. Notably, ICA can also ameliorate bone metabolic disorders in hormone-deficient animal models by restoring gut microbiota homeostasis. For instance, in estrogen-deficiency (ovariectomized) or iron-overload conditions, ICA induces autophagy and reduces oxidative damage, effectively slowing bone loss and improving bone quality [18]. In cartilage regeneration, ICA upregulates hypoxia-inducible factor-1α (HIF-1α) and its downstream pathways, promoting chondrocyte proliferation and the synthesis of matrix components such as type II collagen and proteoglycans. In animal experiments, this leads to higher repair scores (ICRS II) in cartilage defect areas, confirming its potential to promote hyaline cartilage formation [19]. At the molecular level, ICA can inhibit the NF-κB/NLRP3 pathway-mediated inflammatory response, reducing the expression of pro-inflammatory factors like IL-1β and TNF-α and thereby mitigating synovial inflammation and joint degeneration. Furthermore, ICA’s inhibition of the NLRP3 inflammasome and its downstream effector caspase-1 reinforces the mechanistic basis for its efficacy in degenerative diseases such as osteoarthritis [20]. Additionally, ICA exhibits a unique advantage by bidirectionally regulating bone formation and resorption. On the one hand, it promotes the expression of osteogenic factors (Runx2, BMP2, TGF-β1) and increases alkaline phosphatase (ALP) activity, accelerating osteogenesis; on the other hand, it effectively inhibits osteoclastogenesis and bone resorption, helping maintain the dynamic balance between bone formation and bone resorption during bone remodeling [21]. Moreover, recent studies have found that ICA can upregulate the expression of the circadian rhythm gene *BMAL1*, which regulates the osteogenic differentiation capacity of MC3T3-E1 cells and enhances the bone-forming potential of BMSCs in vivo. By activating the *BMAL1*-mediated BMP2/Runx2 signaling module, ICA reveals a potential link between circadian rhythms and bone metabolism regulation [22].

## 2. Pharmacological and Molecular Mechanisms of Icariin

Mechanistic illustration: The diagram illustrates the key regulatory mechanisms of icariin in inflammation modulation, osteogenesis/chondrogenesis, and bone regeneration-associated angiogenesis.

These mechanisms can be divided into three main pathways:

Osteogenesis and chondrogenesis: Icariin promotes the osteogenic differentiation of bone marrow mesenchymal stem cells (BMSCs) through Akt signaling and Wnt/β-catenin activation. It enhances cartilage regeneration by increasing the expression of type II collagen (Col II) and aggrecan while inhibiting matrix degradation and subchondral bone resorption.

Anti-inflammatory effects: Icariin exerts anti-inflammatory effects by inhibiting NF-κB activation and modulating the MAPK cascade (ERK, JNK, p38), thereby reducing the production of IL-1β and TNF-α. In addition, icariin can activate the Nrf2/ARE pathway, increasing antioxidant enzymes such as HO-1, SOD, and CAT, thus rebalancing the inflammatory response.

Anti-inflammatory effects: Icariin exerts significant anti-inflammatory effects by modulating key inflammatory pathways. It inhibits NF-κB activation, reducing the production of pro-inflammatory cytokines such as IL-1β and TNF-α. Furthermore, icariin downregulates the MAPK cascade (ERK, JNK, p38) and NLRP3 inflammasome activation, mitigating inflammatory responses. Additionally, icariin activates the Nrf2/ARE pathway, leading to enhanced expression of antioxidant enzymes such as HO-1, SOD, and CAT, which serve to counteract oxidative stress and rebalance the inflammatory microenvironment, ultimately promoting a more favorable condition for tissue regeneration.

Angiogenesis and bone–vascular coupling: Icariin promotes endothelial cell migration, proliferation, and tube formation by activating ERK and PI3K/Akt signaling, thereby inducing vasodilation and angiogenesis. Moreover, icariin stimulates BMSC osteogenesis and enhances angiogenic signaling to coordinate bone–vascular coupling, facilitating effective bone reconstruction.

In summary, icariin modulates multiple signaling pathways to resolve inflammation, support bone and cartilage regeneration, and promote angiogenesis, ultimately leading to effective bone regeneration.

### 2.1. Anti-Inflammatory Mechanisms

**Figure 1:**
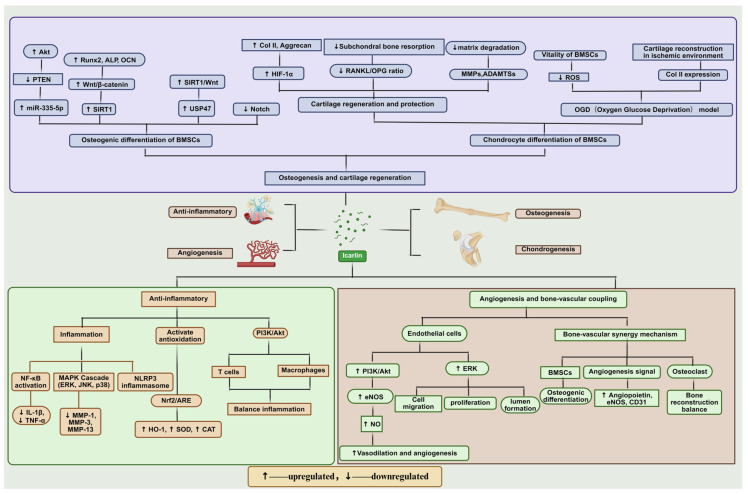
Icariin-mediated regulatory mechanisms in inflammation modulation, osteo-/chondrogenesis, and angiogenesis for bone regeneration.

Inflammation plays a central role in host immune defense and tissue homeostasis. However, when inflammatory responses become persistent or uncontrolled, they can lead to degenerative changes—especially in the musculoskeletal system, where chronic inflammation is a key factor driving tissue damage and functional impairment. Current clinical interventions heavily rely on nonsteroidal anti-inflammatory drugs (NSAIDs) and glucocorticoids, which can relieve inflammation in the short term but cause side effects like immunosuppression and endocrine disorders with long-term use, limiting their utility in chronic conditions [23]. In recent years, ICA has demonstrated significant immunomodulatory activity in various inflammatory disease models. As shown in Figure 1, its main mechanisms include suppressing activation of the NF-κB pathway—thereby downregulating inflammatory mediators such as IL-1β and TNF-α—and acting in concert with autophagy pathways to effectively alleviate chondrocyte apoptosis and matrix degradation. Additionally, ICA can interfere with the MAPK cascade, reducing the activity of matrix-degrading enzymes (MMP-1, MMP-3, MMP-13) while modulating the OPG/RANKL/RANK system to exert anti-osteoclastic effects in an inflammatory bone environment [24].

ICA also exhibits strong efficacy in mitigating oxidative stress. By activating the Nrf2/ARE transcriptional program, it induces antioxidant enzymes, thereby enhancing cellular stress resistance and extracellular matrix stability. In animal models of osteoarthritis and rheumatoid arthritis (RA), ICA’s tissue-protective and anti-inflammatory effects have been repeatedly demonstrated [25]. Notably, ICA has also shown broad therapeutic potential in models of multi-system inflammatory injury. By activating signaling axes such as PI3K/Akt, ICA modulates immune cell function and inflammatory cascades in chronic inflammatory diseases of the respiratory, urinary, reproductive, digestive, and central nervous systems, helping maintain immune homeostasis and suppressing inflammatory mediators. These findings expand its potential utility in managing systemic inflammatory conditions [26].

### 2.2. Osteogenic and Chondrogenic Regenerative Mechanisms

Dynamic coordination between bone regeneration and bone resorption is fundamental for maintaining bone homeostasis; imbalance can lead to degenerative bone diseases like osteoporosis and osteoarthritis [27]. Studies have shown that ICA effectively induces BMSCs to differentiate into osteoblasts. For instance, ICA upregulates miR-335-5p and downregulates its target gene *PTEN*, thereby restoring the osteogenic potential and mineralization capability of BMSCs, suggesting a potential molecular target for osteoporosis therapy [28]. At the molecular level, ICA activates SIRT1, which in turn enhances Wnt/β-catenin signaling to drive osteogenic transcriptional programs. When SIRT1 is inhibited, ICA’s pro-osteogenic effect is significantly weakened, indicating that SIRT1 plays a pivotal role in ICA-mediated osteogenesis [29]. Furthermore, ICA can synergistically activate the USP47/SIRT1/Wnt signaling axis, boosting the efficiency of intracellular osteogenic signaling [30].

In MC3T3-E1 and C3H10T1/2 cell lines, ICA significantly upregulated osteogenic markers like Runx2 and osteocalcin (Ocn), and increased ALP activity. Notably, ICA further enhanced osteogenesis by inhibiting aberrant Notch pathway activation. In osteoporotic animal models, ICA improved bone microarchitecture and increased bone mineral density and mechanical strength, demonstrating a robust bone repair capacity [31]. Meanwhile, ICA also displays unique value in cartilage regeneration and protection. Because articular cartilage is avascular and has limited nutrient supply, its self-repair capacity is low, and it is prone to degeneration under mechanical stress or in an inflammatory microenvironment [32]. ICA can modulate the RANKL/OPG ratio, inhibit subchondral bone resorption, and slow the destruction and degeneration of cartilage matrix [33]. In an oxygen–glucose deprivation (OGD) model simulating an ischemic environment, ICA reduced oxidative stress in BMSCs, enhanced cell viability, and induced the expression of cartilage-specific genes (Col II, aggrecan), indicating its potential value in repairing ischemic cartilage injuries [34].

### 2.3. Angiogenesis and Bone–Vascular Coupling Mechanisms

During bone repair and regeneration, angiogenesis not only supplies oxygen and nutrients but is also critical for stem cell maintenance and regulation of bone metabolism. The metabolic coupling between bone and blood vessels is considered a key step in new bone regeneration and functional reconstruction [35]. As shown in Figure 1, vascular endothelial growth factor (VEGF) drives new vessel formation and maturation by activating branches of the MAPK pathway (MEK1/2 and p38), with p38 serving as a central regulatory node [36].

ICA exhibits unique pro-angiogenic properties. Studies have demonstrated that ICA significantly enhances endothelial cell migration, proliferation, and tube formation—hallmark events of angiogenesis—in various in vitro and in vivo models [37]. As illustrated in Figure 1, this effect mainly involves activation of the PI3K/Akt axis in coordination with ERK and eNOS pathways, leading to increased NO production and improved tissue perfusion. Notably, ICA’s angiogenic activity does not depend on upregulating VEGF expression, suggesting a VEGF-independent mechanism [38]. Overall, ICA can co-activate the MEK/ERK and PI3K/Akt/eNOS pathways, substantially boosting angiogenic potential and supporting tissue repair under ischemic conditions. In bone defect and osteoporosis models, ICA not only promotes BMSC differentiation into osteoblasts but also induces various angiogenic factors and inhibits osteoclast activity, thus coordinating bone regeneration with neovascularization and significantly accelerating bone healing [39].

## 3. Preclinical Evidence for Bone and Cartilage Repair

### 3.1. Animal Model Research Progress

ICA has demonstrated remarkable bone and cartilage regenerative potential in various animal models, involving multi-level regulatory networks such as the expression of osteogenic and chondrogenic genes, remodeling of the local immune microenvironment, activation of key signaling pathways, and intercellular communication.

In a rabbit calvarial defect model, ICA significantly upregulated key osteogenic transcription factors (COL1A2, BMP2, and OSX), enhanced alkaline phosphatase (ALP) activity and type I collagen synthesis, and promoted the recruitment and differentiation of osteoprogenitor cells, creating a biological microenvironment favorable for new bone regeneration [40]. In a hormone-related osteonecrosis model, loading ICA onto a porous tricalcium phosphate (TCP) scaffold to create a composite delivery system enhanced both its osteogenic and angiogenic potential. The system not only markedly improved bone regeneration efficiency but also effectively slowed the progression of bone structural collapse, demonstrating ICA’s multifaceted role in bone tissue reconstruction [41]. Furthermore, in an experimental model of osteonecrosis of the femoral head (ONFH), ICA inhibited osteoblast apoptosis by activating the MAPK/ERK pathway and modulated the OPG/RANK/RANKL signaling axis to inhibit osteoclast activity, thereby maintaining bone metabolic balance [42].

In terms of cartilage regeneration, ICA has shown good efficacy as well. In a New Zealand rabbit knee osteoarthritis (KOA) model, the combined use of ICA-conditioned medium and sulfated chitosan significantly improved the continuity of tissue between the cartilage surface and subchondral bone, optimized the joint interface structure, and alleviated surrounding osteoporotic changes [43]. In a Sprague Dawley rat knee cartilage defect model, ICA loaded on a porous magnesium alloy scaffold upregulated key chondrogenic factors (Wnt5a, β-catenin, Sox9, aggrecan, and Col2a1), promoting cartilage regeneration via activation of the Wnt/β-catenin pathway [44]. Moreover, in a rabbit knee cartilage defect study, ICA co-activated the BMP2/Smad5/Runx2 signaling cascade and, through miR-23a-3p–mediated targeting, promoted BMSC osteogenic differentiation. Applied in conjunction with stem cell transplantation, it significantly enhanced cartilage repair efficacy [45].

### 3.2. Icariin in Different Bone Injury Models

Across various bone injury models, ICA’s multi-target regulatory properties confer significant bone repair potential, involving promotion of bone regeneration, inhibition of bone resorption, and optimization of the bone remodeling microenvironment. In a rat femoral fracture model, local administration of ICA increased bone mineral content and histological scores in a dose-dependent manner; its reparative effect was closely associated with enhanced activities of antioxidant enzymes like glutathione (GSH) and glutathione peroxidase (GSH-Px), as well as an improved cellular redox balance [46]. In infection-related bone defects, ICA can induce osteoclasts to release functional exosomes enriched with miRNA-331-3p by activating the MITF/RAB27A signaling axis. These vesicles target osteoblasts and promote their bone-forming function, revealing a novel repair mode mediated by intercellular communication [47]. In rat femur and tibia fracture models, ICA activates the Wnt/β-catenin and BMP pathways, driving BMSCs to differentiate into osteoblasts and regulating the expression of bone repair-related genes, thereby accelerating structural and functional fracture healing [48]. In bone defect reconstruction studies, controlled-release ICA formulations also show pronounced osteogenic activity, enhancing early bone matrix deposition and improving trabecular organization; at certain doses, their bio-efficacy approaches that of recombinant human bone morphogenetic protein-2 (rhBMP-2) [49].

In a critical-sized calvarial defect model, systemic administration of an ICA-enriched extract effectively induced osteogenesis and reduced osteoclast numbers while also improving trabecular architecture [50]. In a New Zealand rabbit radial defect experiment, loading ICA into a silk fibroin/chitosan/nano-hydroxyapatite ternary composite scaffold combined with BMSC transplantation significantly enhanced bone regeneration and angiogenesis, and it upregulated the expression of key osteogenic proteins such as Runx2 osteocalcin (OCN), VEGF, and Col1α [51]. In models of osteoporosis caused by metabolic disorders, ICA likewise exhibits consistent bone-protective effects. For example, in a diabetic osteoporosis model, ICA upregulated Runx2 and the OPG/RANKL ratio, improving the imbalance between osteogenesis and adipogenesis, potentially through glycemic control and activation of osteogenic pathways [52]. In an ovariectomy-induced bone loss model, ICA stimulated BMSC proliferation, osteogenic differentiation, and mineralization, demonstrating its promise in postmenopausal osteoporosis intervention [53].

In terms of cartilage repair, ICA also exerts strong biological effects. In a mouse cartilage defect model, ICA inhibited the NF-κB/HIF-2α signaling axis, reducing inflammatory mediator release and matrix degradation, while enhancing chondrocyte viability and slowing degenerative progression [54]. To improve its localized efficacy, an HA-Ca-Alg@ICA hydrogel was developed for controlled release; in a rabbit KOA model, it effectively suppressed the Wnt/β-catenin pathway and enhanced cartilage regeneration [55]. Furthermore, in inflammatory joint disease models such as osteoarthritis and rheumatoid arthritis, ICA downregulates NF-κB and uPA expression and upregulates IκB-α protein, thereby reducing synovial inflammation and cartilage structural damage [56]. In an RA model, ICA also inhibited cathepsin K activity, reduced synovial effusion, and slowed cartilage destruction, underscoring its therapeutic potential in immune-mediated inflammatory diseases [57].

## 4. Icariin’s Pharmacological Effects in Multiple Systems: From Target Organ Protection to Pharmacokinetic Optimization

Mechanistic illustration: This figure illustrates the multi-target therapeutic mechanisms of icariin (ICA) across various organs and biological processes. ICA modulates a broad spectrum of signaling pathways involved in inflammation, oxidative stress, apoptosis, fibrosis, and tissue regeneration.

Liver: ICA regulates the AMPK/PGC-1α/GLUT4 axis, enhances fatty acid oxidation, and attenuates oxidative stress, demonstrating potential in the treatment of non-alcoholic fatty liver disease (NAFLD).

Kidneys: ICA targets oxidative stress and the NLRP3 inflammasome to mitigate renal injury and promote tissue repair, thereby contributing to anti-fibrotic and structural recovery effects.

Heart: ICA modulates the NF-κB and ATF6-DR5 signaling pathways to reduce cardiomyocyte apoptosis, fibrosis, and tissue damage, supporting the maintenance and restoration of cardiac function.

Brain: ICA exerts neuroprotective effects via the Nrf2, ERK/MAPK, and caspase-3 pathways, reducing neuroinflammation and promoting neuronal proliferation and differentiation, which may be beneficial in the context of neurodegenerative diseases.

In summary, ICA regulates key pathways related to inflammation, oxidative damage, and apoptosis across multiple organ systems, highlighting its therapeutic potential as a multi-organ protective agent.

### 4.1. Cardioprotective Effects of Icariin and Its Anti-Apoptotic Mechanisms

**Figure 2:**
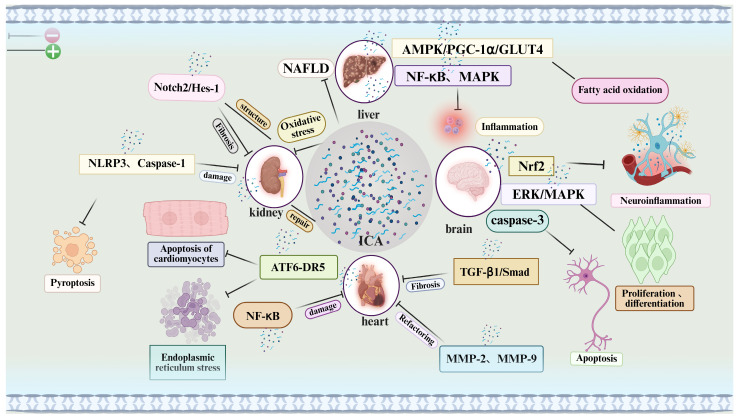
Effects of icariin on major organs.

Apoptosis is a key process in maintaining homeostasis, and its aberrant activation plays an important role in the development of many diseases. Significant cardiomyocyte apoptosis occurs during myocardial infarction, making inhibition of excessive apoptosis an important therapeutic strategy to improve cardiac injury and outcomes. In recent years, ICA has shown notable cardioprotective effects in various animal models, largely attributable to its strong anti-apoptotic activity. Specifically, ICA can effectively suppress isoproterenol-induced apoptosis in neonatal rat cardiomyocytes, maintaining mitochondrial membrane potential stability. As shown in Figure 2, mechanistic studies indicate that this anti-apoptotic effect may be related to the inhibition of the ATF6-DR5 pathway associated with endoplasmic reticulum stress [58]. In addition, ICA possesses broad cardiovascular protective properties, including reducing inflammation, modulating immune function, inhibiting cardiac hypertrophy and fibrosis, and preventing pathological conditions like congestive heart failure and hypertension. In vitro, ICA significantly inhibited angiotensin II–induced hypertrophy and apoptosis in H9c2 cardiomyocytes [59].

Notably, the transforming growth factor-β1 (TGF-β1)/Smad pathway plays a central role in cardiac fibrosis and pathological ventricular remodeling. Animal studies showed that ICA can modulate this pathway, significantly reducing post-myocardial infarction fibrosis and improving ventricular structural and functional remodeling, suggesting potential value in treating cardiac fibrosis. Further cardiac function evaluation revealed that ICA treatment significantly increased left ventricular ejection fraction (LVEF) and fractional shortening (FS), it and decreased left ventricular end-diastolic diameter (LVEDD) and end-systolic diameter (LVESD), improving ventricular performance overall. Additionally, based on the Traditional Chinese Medicine (TCM) theory of “qi deficiency and blood stasis”, some propose that ICA’s cardioprotection may involve improving qi and blood circulation [60].

In a congestive heart failure model, ICA likewise showed significant efficacy. It lowered plasma levels of tumor necrosis factor-α (TNF-α), norepinephrine, angiotensin II, and brain natriuretic peptide, while attenuating isoproterenol-induced left ventricular structural abnormalities and dysfunction. These effects are likely related to ICA’s inhibition of matrix metalloproteinase-2 and -9 (MMP-2, MMP-9) activity and reduction of cardiomyocyte apoptosis, thereby effectively blocking pathological ventricular remodeling. Furthermore, one study noted that ICA can promote the differentiation of mouse pluripotent stem cells into mature cardiomyocytes, expanding its potential applications in cardiac regenerative medicine [61]. In an oxidative stress-induced toxic cardiomyopathy model, ICA still exhibited clear protective effects. It significantly reduced histopathological damage, oxidative injury, and inflammation, and improved hemodynamic parameters, while suppressing ongoing apoptosis and fibrosis. Mechanistically, ICA’s cardioprotection may involve inhibiting NF-κB pathway activation, preventing caspase-3-mediated DNA damage, and activating the NO-cGMP and Nrf2 antioxidant pathways [62].

### 4.2. Icariin Ameliorates Renal Dysfunction via Multi-Target Mechanisms

Chronic kidney disease (CKD) progression is often accompanied by renal tubular epithelial cell injury and interstitial fibrosis, driven by mechanisms such as pyroptosis, epithelial–mesenchymal transition (EMT), and aberrant activation of signaling pathways. Notably, as shown in Figure 2, the Notch signaling pathway—especially the Notch2/Hes-1 axis—plays a key role in fibrosis. In a unilateral ureteral obstruction (UUO)-induced CKD mouse model and a TGF-β1-stimulated HK-2 cell model, ICA significantly inhibited Notch2/Hes-1 pathway activity and downregulated Notch2 mRNA levels, indicating a strong anti-fibrotic potential [63]. Additionally, ICA’s inhibition of pyroptosis has been shown to ameliorate kidney injury. ICA treatment significantly reduced the expression of NLRP3, caspase-1, GSDMD, and IL-1β in the kidneys of UUO rats, alleviating tubular interstitial inflammation and nuclear condensation, suggesting that ICA can exert anti-inflammatory and tissue-protective effects by blocking the pyroptotic process. Simultaneously, ICA markedly suppressed EMT in nephrotic syndrome model rats, evidenced by downregulation of the mesenchymal marker α-smooth muscle actin (α-SMA) and upregulation of the epithelial marker E-cadherin, further confirming its ability to interfere with the EMT process [64]. Regarding renal structural remodeling, ICA significantly improved tubular atrophy, inflammation, oxidative stress, and collagen deposition in the UUO model; restored mitochondrial morphology and function in tubular epithelial cells; and reduced the expression of fibrosis-related proteins, reinforcing its anti-fibrotic efficacy. Consistently, in TGF-β1-induced HK-2 cells, ICA effectively inhibited α-SMA and collagen I expression, supporting its anti-interstitial fibrosis effects at the molecular level [65].

Notably, in an L-NAME-induced acute kidney injury model of pregnancy, ICA demonstrated significant renoprotective effects. It reduced proteinuria, blood urea nitrogen, and serum creatinine levels; increased the expression of the podocyte marker protein nephrin; and inhibited angiotensin II activity, thereby providing podocyte protection and preserving renal function [66]. Recent studies further reveal that ICA positively influences renal stem/progenitor cells. In a 5/6 nephrectomy (subtotal nephrectomy)-induced chronic renal failure model, ICA promoted the expression of stem cell-related genes *Osr1*, *NMP-7, Pax2,* and *WT1* and increased the number of CD133^+^ and/or CD24^+^ renal progenitor cells. Meanwhile, ICA delayed premature differentiation of renal progenitors and inhibited TGF-β-mediated fibrosis, thereby playing an important role in repairing renal structure and function [67]. In summary, through multiple synergistic mechanisms—including inhibition of Notch signaling, EMT, and pyroptosis; modulation of immune and oxidative responses; and activation of stem cell-mediated repair—ICA has demonstrated significant renoprotective potential in both chronic and acute kidney injury models.

### 4.3. Icariin’s Anti-NAFLD Potential via Signaling Pathway Modulation

Liver injury is a common pathological condition that, if not intervened in time, can progress to chronic liver disease and even cirrhosis, seriously threatening human health. Therefore, developing treatments with definite efficacy, high safety, and few side effects is crucial to slowing liver disease progression. Currently, conventional Western medications or integrated traditional Chinese and Western therapies are used for liver diseases, but their efficacy is limited, and especially in patients with compromised liver function, they can cause significant side effects. In this context, natural active compounds have become a research focus, among which the flavonoid icariin (ICA) has attracted attention for its notable hepatoprotective effects. As shown in Figure 2, studies indicate that ICA and its metabolites can exert anti-inflammatory, antioxidant, and anti-fibrotic effects by modulating multiple signaling pathways—such as Akt/GSK3β, PPARα/CPT1a-ACOX1, LKB1/AMPK/ACC, and MAPK—thus providing multi-target protection for the liver. Moreover, given the key role of inflammation in liver injury, the anti-inflammatory mechanisms ICA demonstrates in neurological and renal diseases may also be applicable in liver disease intervention [68].

In recent years, non-alcoholic fatty liver disease (NAFLD) has drawn widespread attention due to its high prevalence, progressive nature, and close association with metabolic syndrome. In a rat model of polycystic ovary syndrome (PCOS) combined with NAFLD, ICA at doses of 40 or 80 mg/kg significantly reduced hepatic lipid accumulation, with the higher dose more effectively enhancing mitochondrial fatty acid oxidation. The mechanism may involve regulation of the CD36–PPAR–CYP4A3 pathway, resulting in reduced fatty acid uptake and decreased lipid deposition, thereby slowing NAFLD progression [69]. Further studies found that ICA markedly improved disordered lipid metabolism and liver histopathology in NAFLD rats by upregulating miR-206; knocking down miR-206 significantly weakened these effects. Mechanistic analysis suggested this involved regulation of the NF-κB and MAPK signaling pathways. Additionally, ICA lowered blood lipid levels, further supporting its potential in NAFLD treatment [70]. In another NAFLD mouse model, ICA (100–200 mg/kg) significantly improved glucose tolerance and insulin sensitivity, while alleviating hepatic histological damage, lipid accumulation, and apoptosis. Mechanistically, ICA downregulated cleaved caspase-3/9, sterol regulatory element-binding protein 1c (SREBP-1c), and diacylglycerol acyltransferase 2 (DGAT2), while upregulating CPT1, AMPKα1, PGC-1α, and GLUT4. Using the AMPK inhibitor dorsomorphin further confirmed that ICA acts via the AMPKα1/PGC-1α/GLUT4 pathway as a core mechanism for improving glucose and lipid metabolism [71]. Notably, some studies have found that ICA may inhibit endoplasmic reticulum (ER) stress and reduce fatty acid-induced lipid synthesis, suggesting a metabolic inhibitory effect. However, in oleic acid/palmitic acid (OA/PA)-induced primary hepatocyte and HepG2 cell models, ICA actually promoted lipid accumulation, indicating it may have bidirectional regulatory properties under different metabolic conditions. Therefore, further investigation of its tissue-specific and metabolism-dependent mechanisms is necessary to fully understand its actions [72]. In summary, by modulating multiple pathways of glucose–lipid metabolism, inflammation, and apoptosis, ICA produces significant improvements in NAFLD models. Its good safety profile and multi-target activity give it potential as a therapeutic candidate for NAFLD and other liver diseases, providing new directions for the prevention and treatment of metabolic liver diseases and a basis for future drug development.

### 4.4. Neuroprotective Mechanisms of Icariin Based on Multi-Target Pathways

Icariin’s neuroprotective effects in neurological disorders have been demonstrated in numerous studies. In a traumatic brain injury model, ICA significantly improved cognitive impairment by enhancing hippocampal cholinergic function, and via an Nrf2-dependent mechanism, it reduced neuroinflammation mediated by glial cells, thereby protecting dopaminergic neurons. Additionally, ICA improved the morphological structure of cortical and hippocampal neurons in aged rats, potentially through activating AMPK, inhibiting mTOR, and enhancing neuronal autophagy [73]. In an Alzheimer’s disease (AD) model, ICA likewise showed significant neuroprotective effects. It improved insulin signaling in the brains of AD mice and increased glucose transporter expression, markedly reducing Aβ deposition and abnormal tau hyperphosphorylation. By maintaining insulin signaling homeostasis and glucose metabolic balance, ICA preserved neuronal and synaptic integrity, effectively improving learning and memory. With its low toxicity, ICA is considered a promising novel therapeutic candidate for AD [74]. Beyond AD, ICA has exhibited notable efficacy in models of cerebral ischemia, Parkinson’s disease, depression, and multiple sclerosis, and it may slow nervous system aging. Furthermore, *Epimedium* extracts have neuroprotective effects against ionizing radiation, preventing radiation-induced impairment of neurogenesis, which broadens ICA’s prospects in neuropsychiatric disorders and radiation-induced brain injury.

In vitro, ICA also promotes neural stem cell (NSC) function. By regulating key factors such as cyclin D1 and p21, ICA enhanced the proliferation and differentiation of human and rat hippocampal NSCs. Notably, in aged animal models, ICA stimulated quiescent NSCs to enter an active state, boosting their proliferation, survival, and migration, and it induced their differentiation into neuronal lineages (Figure 2). In addition, ICA activated the ERK/MAPK pathway to further promote NSC self-renewal and neurogenesis [75]. ICA’s neuroprotective mechanisms have been further confirmed in various disease models. For example, in aged rats, ICA activated autophagy in the cortex and hippocampus via the AMPK/mTOR/ULK1 pathway, thereby mitigating age-related cognitive decline. In a neonatal mouse hypoxic–ischemic brain injury (HIBD) model, ICA pretreatment significantly inhibited apoptosis and reduced neural tissue damage, with the mechanism likely involving upregulation of estrogen receptors α (ERα) and β (ERβ), both of which are key in regulating apoptosis and autophagy. Further research found that neuroprotective miR-7-1 can, by enhancing the activity of estrogen receptor agonists (e.g., estradiol), synergistically reduce neuronal apoptosis after spinal cord injury, which may further augment ICA’s neuroprotective effects [76]. Moreover, during hypoxia–ischemia-induced neural injury, activation of caspase-3 is a central mechanism of neuron death. TUNEL staining and Western blot analysis showed that ICA pretreatment significantly inhibited the expression of cleaved caspase-3, suggesting that it can attenuate ischemic brain injury via anti-apoptotic mechanisms [77]. In summary, by modulating multiple key signaling pathways (such as AMPK, Nrf2, ERK/MAPK, and AMPK/mTOR/ULK1), ICA exerts anti-inflammatory, anti-apoptotic, pro-NSC, and autophagy-enhancing effects, demonstrating significant efficacy in the prevention and treatment of neurodegenerative diseases and broad prospects for clinical translation.

### 4.5. Cytotoxicity of Icariin in Cell Models

Icariin has shown significant anti-tumor activity in various cancer cell models, and its cytotoxicity is achieved through multiple mechanisms acting in concert, including inducing cell cycle arrest, activating oxidative stress responses, and triggering programmed cell death. However, due to ICA’s poor water solubility and limited oral bioavailability, its clinical translation is somewhat restricted. To overcome these pharmacokinetic limitations, researchers have developed a variety of nanotechnology-based delivery systems, such as plant-derived carriers and cubosome lipid nanoparticles, to enhance its dissolution rate, cellular uptake efficiency, and targeting specificity. For example, an ICA plant carrier optimized via Box–Behnken response surface design showed significant cytotoxicity in OVCAR-3 ovarian cancer cells. The optimized formulation had an ideal particle size distribution and stable morphology, and it exhibited excellent in vitro sustained-release characteristics. Further in vitro results demonstrated that this formulation significantly induced G1 and G2/M phase cell cycle arrest, with Annexin V staining showing a markedly increased proportion of early and late apoptotic cells. Meanwhile, it caused disrupted mitochondrial membrane potential, increased caspase-3 activity, and elevated ROS levels, suggesting it induces apoptosis through a mitochondria-dependent intrinsic pathway, thereby enhancing anti-cancer efficacy [78].

Similarly, in SKOV-3 and Caov-3 ovarian cancer cell models, an ICA-loaded cubosome formulation (ICA-Cubs) exhibited much stronger cytotoxicity than free ICA, with the IC_50_ decreasing from 40.1 μM to 11.2 μM, and it showed lower toxicity toward non-tumorigenic endothelial cells (EA.hy926), reflecting good targeting selectivity. Mechanistic studies further indicated that ICA induces G0/G1 and G2/M phase arrest and activates apoptotic signaling by upregulating p53 and caspase-3 expression. It also significantly promotes ROS generation, inducing programmed cell death via the p53/caspase-3 axis without causing notable necrosis. Additionally, ICA upregulates TNF-α and inhibits NO production, suggesting it may modulate the tumor microenvironment to enhance anti-cancer efficacy [79]. Notably, ICA’s synergistic pro-apoptotic effect in combination with chemotherapy has also drawn attention. In a doxorubicin (DOX)-resistant MG-63/DOX osteosarcoma cell model, ICA enhanced DOX-induced apoptosis, with higher apoptosis rates accompanied by significantly increased cleaved PARP and caspase-3 fragments. To further clarify the pathway, researchers measured caspase-9 levels and found that ICA downregulated procaspase-9 (47 kDa), suggesting its pro-apoptotic effect is achieved via a mitochondria-mediated intrinsic apoptotic pathway. Additionally, the PI3K/Akt pathway—an upstream negative regulator of caspase-9—was identified as one of the key targets of ICA [80]. Besides exhibiting strong toxicity in cancer cells, ICA displays good protective effects in non-tumor systems. For instance, in a cisplatin-induced oxidative stress model in HEK-293 cells, ICA pretreatment effectively reduced oxidative stress and inflammatory damage. Its mechanisms included lowering malondialdehyde (MDA) and ROS levels, increasing GSH content, inhibiting NF-κB phosphorylation and nuclear translocation, thereby reducing the expression of IL-1β, TNF-α, and inducible nitric oxide synthase (iNOS). Moreover, by downregulating Bax and caspase-3/9 and upregulating Bcl-2, it exerted anti-apoptotic effects. The PI3K-specific inhibitor LY294002 significantly reversed ICA’s protective effects, further verifying that its actions depend on the PI3K/Akt pathway [81]. In another study, ICA was also shown to significantly improve the post-thaw survival and function of human umbilical cord mesenchymal stem cells (hUC-MSCs). Specifically, ICA enhanced cell adhesion, reduced LDH release, repaired the F-actin cytoskeleton, maintained mitochondrial membrane potential (ΔΨm) stability, and improved mitochondrial function. Importantly, ICA itself showed no cytotoxicity in this model; instead, by reducing oxidative stress and upregulating heat shock protein expression, it significantly preserved stem cell phenotype and function. This provides theoretical and practical support for developing ICA as a cryoprotective additive in cell cryopreservation [82]. In summary, ICA exhibits potent multi-mechanistic cytotoxicity in tumor cells and synergistic effects in combination therapy, while in normal cell systems it shows protective properties and biosafety. These findings lay the foundation for its broad application in cancer therapy and stem cell engineering.

### 4.6. Pharmacokinetics of Icariin: Exploration of In Vivo Half-Life

Flavonoids generally have low oral bioavailability, which is considered a major obstacle limiting their clinical translation. As a typical example of this class, icariin (ICA) has an oral bioavailability of around 12%, with limited absorption efficiency, severely constraining the full exertion of its pharmacological effects and therapeutic potential. Its absorption barrier is mainly related to its poor water solubility, low membrane permeability, and slow dissolution rate in body fluids. Studies using ultra-high-performance liquid chromatography–tandem mass spectrometry (UPLC-MS/MS) to quantify ICA in rat plasma after oral administration showed no significant differences in major pharmacokinetic parameters between male and female rats, and overall low plasma drug concentrations at all time points, indicating insufficient oral absorption. ICA’s maximum plasma and tissue concentrations (C_max_) usually occur 0.5–1 h after administration and then decline rapidly within 4 h, reflecting quick absorption and elimination with no obvious accumulation. Tissue distribution analysis further showed that ICA mainly accumulates in the liver, lungs, and reproductive organs, indicating some tissue targeting [83]. Further research found that the absolute bioavailability of ICA in rats is only 0.1–1.8%, indicating extremely low systemic exposure. Its C_max_ is often reached within 30–60 min of dosing; although absorption is rapid, utilization is poor, mainly due to limited intestinal absorption and a pronounced hepatic–intestinal first-pass effect. ICA is primarily metabolized via hepatic glucuronidation and sulfation and is eliminated in urine and feces. Notably, its pharmacokinetics exhibit nonlinear characteristics, possibly related to absorption saturation, modulation of metabolic enzyme activity, or changes in tissue distribution [84]. Regarding elimination, ICA’s plasma half-life (t_1_/_2_) is generally 1.2–3.5 h, indicating fast clearance and a limited duration of action; it also shows poor stability in ex vivo serum, further limiting its sustained effects. Tissue distribution results indicate that ICA accumulates mainly in the liver, kidneys, intestines, and lungs, suggesting the possibility of enterohepatic circulation. Additionally, under the action of intestinal microbiota, ICA can be transformed into more active metabolites such as icaritin, indicating certain prodrug characteristics [85]. To overcome ICA’s low bioavailability, various novel drug delivery systems have been developed in recent years, including solid lipid nanoparticles (SLNs), nanoemulsions, polymeric micelles, liposomes, and self-emulsifying drug delivery systems (SEDDSs). These formulations significantly improve ICA’s water solubility and stability, enhance its gastrointestinal absorption, and prolong its circulation time. For example, ICA-loaded poly(lactic-co-glycolic acid) (PLGA) nanoparticles significantly prolonged ICA’s plasma retention time in animal experiments and improved its penetration into brain tissue [86]. In addition, forming an inclusion complex of ICA with hydroxypropyl-β-cyclodextrin greatly increased its solubility and membrane permeability. Intestinal perfusion studies showed that this complex had better absorption than conventional β-cyclodextrin, possibly due to enhanced solubilization and inhibition of P-glycoprotein efflux. Another study reported that after intravenous administration using lyophilized stealth SLNs, ICA’s t_1_/_2_ was extended from 0.21 h to 1.4 h, and AUC_0→∞_ increased from 0.82 mg·h/L to 3.34 mg·h/L, dramatically increasing systemic exposure [87]. In summary, although ICA possesses a variety of biological activities, its low oral bioavailability and rapid clearance in vivo remain key issues limiting its clinical application. Future research should focus on nanotechnology-based efficient delivery systems, optimization of prodrug strategies, and construction of targeted absorption platforms, so as to enhance its systemic exposure, prolong the duration of effect, and improve tissue penetration, thereby maximizing its clinical therapeutic potential.

## 5. Clinical Applications and Translational Potential

### 5.1. Challenges in Transition from Preclinical to Clinical

In vitro, ICA has significant potential to induce human bone marrow mesenchymal stem cells (hBMSCs) to differentiate into the osteogenic lineage, with a concentration-dependent effect. The optimal range is considered 10^−9^ to 10^−5^ M, beyond which cytotoxicity may occur [88]. However, current studies mainly rely on in vitro co-culture systems that cannot fully simulate the complex bone metabolic environment in humans, making it difficult to directly translate these results to clinical applications [89]. ICA exhibits markedly different optimal effective concentrations across various experimental systems, indicating an undefined therapeutic window that may hinder precise clinical dosing. Although studies have noted that ICA can promote bone repair by activating the BMP2/Smad5/Runx2 axis, a systematic understanding of its role in multi-cellular interactions and signaling networks is still lacking [90]. Therefore, further elucidating the pathways by which it participates in bone reconstruction could provide theoretical support for precise clinical interventions.

From a pharmacokinetic perspective, ICA suffers from low bioavailability, rapid metabolism, and poor stability in vivo. It is necessary to improve its pharmacokinetic profile through structural modification, smart delivery platforms, or targeted modifications, to increase its exposure levels and consistency of biological effects. Additionally, its performance varies across different formulations; issues of formulation standardization and dose controllability are also critical technical barriers to clinical translation [91]. Systemic conditions like diabetes significantly disrupt BMSC function, resulting in reduced proliferation, decreased differentiation potential, and increased apoptosis, especially in the presence of osteoporosis. Some studies suggest ICA can modulate diabetes-related osteogenic dysfunction, but a thorough evaluation of its long-term safety and stability in high-glucose microenvironments is needed to facilitate its clinical use under such pathological conditions [92]. In degenerative bone and joint diseases like knee osteoarthritis, ICA has shown effects in improving cartilage structure and slowing pathological degeneration. Yet its path to clinical application is still limited by suboptimal pharmacokinetics, lack of long-term safety data, and unclear effective dose ranges. Establishing a comprehensive pharmacokinetic–pharmacodynamic research framework will be key for its further development [93]. Most current research on ICA involves short-term observations, and a comprehensive assessment of its long-term efficacy and potential toxicity is lacking. Meanwhile, the multi-pathway synergistic mechanisms of ICA in complex tissue environments have not been fully elucidated, hindering its deeper application in chronic bone disorders. Therefore, systematic investigation of its long-acting mechanisms, safety margins, and signal integration will be important to enhance its clinical viability [94].

### 5.2. Clinical Research on ICA’s Therapeutic Potential: Efficacy and Mechanisms

Network pharmacology and molecular docking studies have systematically explored ICA’s potential mechanisms for treating osteoarthritis (OA). The findings suggest that ICA can regulate cell apoptosis, inflammatory cytokine release, and chondrocyte differentiation, indicating immunomodulatory and analgesic effects, although its core targets and pathways require further validation [95]. In osteoporosis research, ICA maintains bone metabolic homeostasis by activating the PI3K-Akt pathway and networking with MAPK, JNK, NF-κB and others, thereby coordinately regulating bone metabolism. Additionally, ICA upregulates osteogenic markers like osteopontin, osteocalcin, and type I collagen; promotes BMP2 and BMP4 production; and increases the osteogenic transcription factor osterix (Osx). This leads to enhanced expression of pro-osteogenic genes such as RUNX2 and ALP, and through modulating the OPG/RANKL axis, it inhibits bone resorption [96,97]. Although in animal models ICA exhibits anti-inflammatory, antioxidant, and osteogenic activities, clinical research is still sparse. Key pharmacokinetic parameters, safe dosage, and therapeutic windows remain undefined, constituting major obstacles to clinical translation [98]. In rheumatoid arthritis (RA) and inflammatory bowel disease (IBD) models, ICA modulates the NF-κB and MAPK pathways to exert anti-inflammatory effects. However, evidence in humans is limited; large-sample, multicenter prospective clinical trials are needed to clarify its safety, efficacy, and range of indications [99].

ICA can also alleviate RA symptoms by inhibiting osteoclast markers (e.g., β3 integrin, cathepsin K, MMP-9) and suppressing IL-17 production via Th17 cells and STAT3, thereby adjusting the OPG/RANKL balance. Moreover, ICA upregulates the miR-223-3p/NLRP3 pathway to induce apoptosis in rheumatoid arthritis fibroblast-like synoviocytes (RA-FLS), suggesting that miR-223-3p may be a potential target of ICA in RA therapy [100]. In postmenopausal osteoporosis and other bone metabolic disorder models, ICA engages in osteogenic signaling (including via non-coding RNAs like miR-23a) to induce BMSC osteogenic differentiation and improve bone mass and mechanical properties, indicating good potential for clinical translation [101]. Studies have reported that at 20 μM, ICA significantly upregulates osteogenic factors and acts synergistically with BMP2. At 38.4 μM, its osteogenic effect exceeded that of BMP2 at 0.8 μg/mL, suggesting ICA could potentially substitute for traditional osteogenic factors [102]. ICA also enhances Cbfa1 expression and promotes OPG transcription, and in ovariectomized rats, it dose-dependently increased osteocalcin levels [103]. In models of osteoporosis caused by hormonal imbalance and metabolic disturbances, ICA activates PI3K-Akt and Nrf2 pathways, regulating bone resorption and formation processes and demonstrating broad-spectrum bone-protective effects [104]. In an alcohol-induced osteoporosis model, ICA upregulated BMP2 and activated Smad signaling, thereby influencing key factors like Osx and Runx2 to promote bone regeneration [105]. Animal experiments confirm that ICA can significantly increase bone density and improve bone microarchitecture, but human confirmation is still lacking. To date, no randomized controlled trials have examined its effects on fracture healing or bone defect repair, and its osteogenic potential remains to be verified clinically [106]. Overall, while ICA’s anti-inflammatory, pro-osteogenic, and signaling–regulatory mechanisms in bone disorders have been demonstrated experimentally, its clinical translation is hampered by unclear pharmacokinetic and toxicological profiles and a lack of robust clinical evidence. Future efforts should focus on well-designed randomized controlled trials to comprehensively evaluate its safety, efficacy, and indications, propelling its development as a novel bone repair therapeutic [107].

## 6. Current Challenges and Future Prospects

### 6.1. Bioavailability and Pharmacokinetic Limitations

ICA has shown therapeutic potential in various disease models, but its clinical use is impeded by significant pharmacokinetic hurdles, particularly an extremely low oral absorption efficiency [108]. Mainly due to its poor water solubility and inadequate membrane permeability, ICA is classified as a Biopharmaceutics Classification System (BCS) Class IV drug. In the gastrointestinal tract, ICA’s stability is limited and it is absorbed slowly via passive diffusion in the small intestine, resulting in a very low fraction of the parent compound reaching systemic circulation [109]. Pharmacokinetic studies further indicate that ICA is rapidly metabolized by hepatic enzymes and gut microbiota into icariin and related derivatives; although these metabolites have some pharmacological activity, their systemic exposure is low, limiting the duration of drug action. Additionally, ICA predominantly accumulates in the liver and kidneys and is excreted via bile, with minimal parent drug detected in urine and feces, reflecting rapid metabolism and clearance. Individual differences in P-glycoprotein expression, gut microbiota composition, and liver enzyme activity also significantly affect ICA’s disposition [110]. To address these limitations, researchers have proposed various delivery strategies and formulation improvements, including phospholipid complexes, solid dispersions, cyclodextrin inclusion complexes, and nano-carrier platforms. Phospholipid complexes, due to their membrane affinity and amphiphilic nature, markedly improve ICA’s solubility and intestinal permeability, reportedly increasing its bioavailability by 3.4-fold compared to unformulated ICA [111]. Moreover, inclusion complexes of ICA with β-cyclodextrin or hydroxypropyl-β-cyclodextrin have been shown to significantly enhance its dissolution and epithelial penetration [112]. In summary, ICA exhibits a characteristically unfavorable pharmacokinetic profile in terms of absorption, metabolism, and elimination. Future efforts should combine targeted delivery systems, carrier design, and absorption-enhancing strategies to collectively raise its systemic exposure and improve clinical translation efficiency.

### 6.2. Quality Control and Standardization Issues

As the representative bioactive component of *Epimedium* herbs, ICA content is widely used to gauge the quality and efficacy of the raw material. However, current quality evaluation systems cannot fully account for the diversity of sources and the complexity of constituents. Studies have shown that ICA content in the herb is affected by factors such as geographic origin, species, harvest season, and storage conditions, leading to inconsistencies in composition and batch-to-batch stability and thus impacting reproducibility of clinical outcomes [113]. Additionally, ICA’s structure is similar to other flavonoid constituents in *Epimedium*, making it prone to interference during extraction and chromatographic separation, which reduces quantification accuracy. Its measured content is also influenced by extraction parameters (e.g., solvent type, temperature, extraction time), causing fluctuations in active ingredient levels in final products. The low level of standardization makes it difficult to support consistent pharmacodynamic evaluation [114]. Currently, the Chinese Pharmacopoeia uses HPLC to quantify ICA content; although practical, using a single component as the quality control marker fails to reflect the overall synergistic efficacy of multi-component formulations [115]. To address this, researchers have introduced modern analytical techniques like LC-MS/MS, fingerprint profiling, near-infrared spectroscopy, and UPLC-QTOF-MS to construct a more comprehensive and systematic quality evaluation approach [116]. For more refined and scientific quality control, a multi-component collaborative control model should be established, covering the entire process from raw material to final product. By combining chemometric methods with high-resolution spectroscopic techniques, it is possible to effectively discern patterns in active constituents and reinforce full-chain traceability from harvesting and storage to processing. Such efforts will push the quality control system for *Epimedium*-derived products toward greater standardization and systematization [117].

### 6.3. Application Expansion and Technological Frontiers

To enhance ICA’s active exposure and tissue targeting, intelligent delivery platforms have been widely explored in recent years, including liposomes, solid lipid nanoparticles (SLNs), polymer microspheres, hydrogels, and pH-sensitive nanosystems. These delivery strategies improve its stability and dissolution in vivo, significantly prolong its plasma half-life, and enhance tissue affinity and duration of efficacy. In particular, for bone-targeted delivery, controlled-release systems based on biodegradable materials have shown favorable release profiles and targeted accumulation in animal studies [118]. Building on this, stimulus-responsive delivery platforms represent a cutting-edge approach. By enabling drug release triggered by inflammatory or acidic microenvironments, ICA can achieve enhanced local concentration and site-specific delivery in target tissues, extending its local therapeutic duration while reducing systemic side effects. For example, integrating modified liposomes with functional nanoparticles not only improved ICA’s dissolution but also increased cellular uptake and targeted delivery efficiency [119]. Furthermore, incorporating ICA into biomaterial scaffold systems offers new avenues for tissue engineering applications. Biocompatible materials such as chitosan–hydroxyapatite composites can stably load and release ICA in a sustained manner while improving the bone microenvironment and promoting new bone regeneration, thus providing strong support for bone tissue repair [120]. Combination therapy strategies are another promising direction to boost ICA’s therapeutic efficacy. Research indicates that combining ICA with other bioactive molecules (e.g., anti-inflammatory drugs, antioxidants, bone metabolism regulators) can concurrently activate multiple critical pathways, producing synergistic enhancements that show promise in models of degenerative diseases and chronic inflammation [121]. In summary, through the development of multifunctional smart carriers, composite material scaffolds, and combined treatment strategies, it is possible to overcome ICA’s inherent pharmacokinetic limitations and provide integrated solutions for targeted therapy and clinical translation, thereby laying a robust foundation for ICA to realize greater potential in the treatment of bone diseases and related chronic conditions.

## 7. Summary of Research Progress

Icariin (ICA), an active flavonoid extracted from *Epimedium*, has shown significant multi-target effects and potential in promoting bone injury repair. Based on current research, ICA facilitates bone healing through the following mechanisms: Firstly, in inflammation modulation, ICA has notable immunoregulatory and anti-inflammatory effects. It can inhibit the excessive activation of inflammatory pathways like NF-κB and modulate the MAPK cascade, thereby reducing the production of pro-inflammatory mediators (e.g., IL-1β, TNF-α). Meanwhile, it activates the Nrf2/ARE antioxidant pathway, increasing the expression of enzymes such as HO-1 and SOD, to rebalance and resolve inflammation. Effective control of the inflammatory microenvironment creates favorable conditions for subsequent tissue regeneration. Secondly, in osteogenesis and chondrogenesis, ICA directly promotes osteoblast differentiation and matrix production while also protecting cartilage structure. Specifically, ICA activates signals like Akt and Wnt/β-catenin to drive osteogenic differentiation of mesenchymal stem cells, and it upregulates osteogenic factors (e.g., Runx2, BMP2) to enhance bone matrix deposition. In chondrocytes, ICA upregulates HIF-1α and its downstream pathways, increasing the synthesis of type II collagen and proteoglycans, thus stimulating cartilage matrix regeneration. Notably, ICA also inhibits excessive subchondral bone resorption and matrix degradation, preserving osteochondral interface integrity. Interestingly, ICA simultaneously promotes bone regeneration and suppresses osteoclast activity, maintaining a dynamic balance between osteogenesis and bone resorption during bone remodeling. This multi-target bidirectional regulation helps improve the quality of bone repair. Thirdly, promoting angiogenesis is another key action of ICA in bone repair. Adequate blood supply is critical for bone regeneration. ICA significantly enhances endothelial cell migration, proliferation, and tube formation by activating ERK and PI3K/Akt/eNOS signaling, leading to increased nitric oxide (NO) release and tissue perfusion, and thereby stimulating new blood vessel formation. Importantly, ICA couples osteogenesis with angiogenesis: while promoting osteoblastic differentiation, it concurrently upregulates various angiogenic factors, achieving coordinated regulation of bone reconstruction and neovascularization and accelerating the healing of bone defects. Thus, ICA supports regenerating bone tissue by acting on both osteogenic and angiogenic pathways, ensuring nutritional supply and structural integration for the new bone.

It must be emphasized that ICA’s effects are not confined to the local bone environment; its multi-organ actions also contribute to bone repair. In the cardiovascular system, ICA modulates the NF-κB and ATF6-DR5 pathways to reduce cardiomyocyte apoptosis and cardiac fibrosis, protecting heart function. In the liver, ICA activates the AMPK/PGC-1α/GLUT4 pathway to enhance fatty acid oxidation and reduce oxidative stress, which is beneficial in metabolic conditions such as fatty liver disease. In the kidneys, ICA inhibits NLRP3 inflammasome-mediated inflammation, mitigating renal injury and fibrosis. In the nervous system, ICA exerts neuroprotective effects through pathways like Nrf2 and ERK/MAPK, reducing neuroinflammation and promoting neuron survival and functional recovery. These multi-organ effects suggest that ICA can improve the systemic environment during bone healing—on one hand, preventing dysfunction of vital organs (heart, liver, kidneys, brain) from adversely affecting bone regeneration, and on the other, providing systemic support that synergistically promotes tissue repair. This broad-spectrum, multi-target profile highlights ICA’s advantage as a regenerative enhancer.

Leveraging the above mechanisms, ICA has shown great potential in tissue engineering and regenerative medicine applications. Researchers have incorporated ICA into biomaterial scaffolds and hydrogels for bone tissue engineering. The results indicate that ICA-loaded scaffolds can achieve stable drug encapsulation and controlled release, effectively improving the local bone microenvironment and stimulating osteogenesis and angiogenesis, thereby significantly enhancing bone defect repair. For example, combining ICA with biocompatible materials (such as chitosan–hydroxyapatite composites) enables sustained ICA release and accelerates new bone regeneration; when used alongside stem cell transplantation, ICA boosted the osteogenic differentiation and angiogenic capacity of the cells, achieving repair outcomes comparable to those with recombinant growth factors. These explorations demonstrate the benefits of integrating ICA’s multi-pathway regulatory effects with biomaterials, offering new solutions for bone tissue regeneration.

Despite ICA’s unique multi-target, multi-pathway advantages in bone injury repair, current research still faces several limitations and challenges. First, ICA’s pharmacokinetic properties are suboptimal: oral bioavailability is only around 12%, and poor absorption and pronounced first-pass metabolism result in insufficient effective concentrations in vivo. Additionally, ICA is rapidly cleared with a short half-life, which constrains its therapeutic efficacy. Second, as a plant-derived natural product, ICA preparations lack unified standards in extraction and formulation; consequently, different studies or products may vary in active content and dosing, and quality control is not yet standardized. These issues affect the reproducibility of results and the controllability of clinical use. Furthermore, data on its long-term safety and stability under complex pathological conditions are still lacking. Such challenges must be addressed in future research.

In conclusion, to fully exploit icariin’s multi-system and multi-target benefits in bone injury repair, future studies should focus on several aspects. (1) Optimize delivery systems to increase ICA’s effective concentration and duration of action in vivo: for instance, employ nanotechnology to develop targeted delivery vectors and smart controlled-release platforms to achieve targeted accumulation and sustained release of ICA at bone injury sites, thereby improving its bioavailability and prolonging its therapeutic effects. (2) Strengthen systematic pharmacological and safety evaluations and gradually advance to clinical trials: establish sound pharmacokinetic–pharmacodynamic models and gathering evidence-based data to determine the optimal dosing regimen of ICA in humans, while monitoring long-term safety. (3) Explore ICA’s applications under special pathological conditions: investigate its bone-repair effects and mechanisms in contexts such as diabetes, age-related osteoporosis, or radiation-induced bone injury, and assess its synergistic effects with standard therapies, to expand its indications. Through the comprehensive implementation of these strategies, ICA is expected to overcome its current limitations and emerge as a safe and effective multi-target promoter of bone repair in the field of tissue engineering and regenerative medicine, playing a greater role in orthopedic clinical practice and the management of related chronic diseases.

## Data Availability

No new data were created or analyzed in this study. Data sharing is not applicable to this article.
